# Supplementary material for: Dietary protein increases T-cell-independent sIgA production through changes in gut microbiota-derived extracellular vesicles
Source: Nat Commun. 2022 Jul 27;13:4336. doi: 10.1038/s41467-022-31761-y (PMC9329401; doi:10.1038/s41467-022-31761-y)
Supplement: Supplementary file 1 — Supplementary Information [file 41467_2022_31761_MOESM1_ESM.pdf]

**Supplementary Table 1 – Detailed composition of the experimental diets used in the study**

Diet formulation provided as gram of ingredients per 100g for each of the 10 diets used in the study. The diets are isocaloric at 14.5 MJ/kg with the following net metabolizable energy (NME) assigned: casein (13.3 kJ/g; 3.2 kCal/g), L-methionine (18 kJ/g; 4.3 kCal/g), Canola oil (36.6 kJ/g; 8.7 kCal/g), Wheat starch (14.13 kJ/g; 3.4 kCal/g), Dextrinized Starch (14.6 kJ/g; 3.49 kCal/g), Sucrose 14.92 kCal/g and cellulose was given a NME of 0 kCal/g.

| | | **Diet 1** | **Diet 2** | **Diet 3** | **Diet 4** | **Diet 5** | **Diet 6** | **Diet 7** | **Diet 8** | **Diet 9** | **Diet 10** |
|---|---|---|---|---|---|---|---|---|---|---|---|
| | **%P** | 60 | 5 | 5 | 33 | 33 | 5 | 14 | 14 | 42 | 24 |
| | **%C** | 20 | 75 | 20 | 47 | 20 | 47 | 29 | 57 | 29 | 38 |
| | **%F** | 20 | 20 | 75 | 20 | 47 | 48 | 57 | 29 | 29 | 38 |
| **INGREDIENTS** | | | | | | | | | | | |
| **Protein** | **Casein** | 64.87 | 5.04 | 5.04 | 35.50 | 35.50 | 5.04 | 14.83 | 14.83 | 45.29 | 25.71 |
| | **L-Methionine** | 0.30 | 0.30 | 0.30 | 0.30 | 0.30 | 0.30 | 0.30 | 0.30 | 0.30 | 0.30 |
| **Fat** | **Canola Oil** | 7.91 | 7.91 | 29.65 | 7.91 | 18.58 | 18.98 | 22.53 | 11.46 | 11.46 | 15.03 |
| **Carbohydrate** | **Wheat Starch** | 12.59 | 47.87 | 12.60 | 29.91 | 12.60 | 29.91 | 18.37 | 36.34 | 18.37 | 24.13 |
| | **Dextrinized Starch** | 4.10 | 15.57 | 4.10 | 9.73 | 4.10 | 9.73 | 5.98 | 11.82 | 5.98 | 7.85 |
| | **Sucrose** | 3.11 | 11.81 | 3.11 | 7.38 | 3.11 | 7.38 | 4.54 | 8.97 | 4.54 | 5.96 |
| **Minerals** | **$CaCO_3$** | 1.31 | 1.31 | 1.31 | 1.31 | 1.31 | 1.31 | 1.31 | 1.31 | 1.31 | 1.31 |
| | **NaCl** | 0.26 | 0.26 | 0.26 | 0.26 | 0.26 | 0.26 | 0.26 | 0.26 | 0.26 | 0.26 |
| | **AIN93 Trace Minerals** | 0.14 | 0.14 | 0.14 | 0.14 | 0.14 | 0.14 | 0.14 | 0.14 | 0.14 | 0.14 |
| | **$KH_2PO_4$** | 0.69 | 0.69 | 0.69 | 0.69 | 0.69 | 0.69 | 0.69 | 0.69 | 0.69 | 0.69 |
| | **$K_2SO_4$** | 0.16 | 0.16 | 0.16 | 0.16 | 0.16 | 0.16 | 0.16 | 0.16 | 0.16 | 0.16 |
| | **KCl** | 0.25 | 0.25 | 0.25 | 0.25 | 0.25 | 0.25 | 0.25 | 0.25 | 0.25 | 0.25 |
| | **$C_5H_{14}ClNO$** | 0.25 | 0.25 | 0.25 | 0.25 | 0.25 | 0.25 | 0.25 | 0.25 | 0.25 | 0.25 |
| **Vitamins** | **AIN93 vitamins** | 1.00 | 1.00 | 1.00 | 1.00 | 1.00 | 1.00 | 1.00 | 1.00 | 1.00 | 1.00 |
| **Cellulose** | **Cellulose** | 3.06 | 7.44 | 41.15 | 5.21 | 21.75 | 24.60 | 29.39 | 12.23 | 10.00 | 16.97 |

**Supplementary Table 2 – Statistical output for mixture models relating to sIgA**

| Model (Scheffé Polynomials) | Akaike Information Criterion | Degrees of freedom | |
|---|---|---|---|
| 1 | 1168.363 | 4 | |
| 2 | 1168.552 | 7 | |
| 3 | 1156.861 | 11 | |
| 4 | 1170.262 | 8 | |
| Null | 1181.324 | 2 | |
| **Model 1 Coefficients** | | | |
| **Components** | **Estimate (Std. Error)** | **t value** | **P(>\|t\|)** |
| Protein | 2094.2 (264.7) | 7.913 | 2.05e−11 |
| Fat | 517.6 (214.5) | 2.413 | 0.018334 |
| Carbohydrate | 868.8 (220.6) | 3.939 | 0.000185 |
| | | | |
| **Adjusted R-squared** | 0.8076 | | |
| **p-value** | < 2.2e−16 | | |

**Supplementary Table 3 – Statistical output for mixture models relating to plasma IgA**

| Model (Scheffé Polynomials) | Akaike Information Criterion | Degrees of freedom | |
|---|---|---|---|
| 1 | 1160.604 | 4 | |
| 2 | 1142.835 | 7 | |
| 3 | 1149.336 | 11 | |
| 4 | 1144.762 | 8 | |
| Null | 1171.433 | 2 | |
| **Model 2 Coefficients** | | | |
| **Components** | **Estimate (Std. Error)** | **t value** | **P(>\|t\|)** |
| Protein | 1244.4 (600.3) | 2.073 | 0.041706 |
| Fat | 2042.4 (371.3) | 5.501 | 5.31e−07 |
| Carbohydrate | 2110.2 (375.5) | 5.619 | 3.30e−07 |
| Protein:Fat | −3432.3 (1666.8) | −2.059 | 0.043043 |
| Protein:Carbohydrate | −4738.6 (1667.2) | −2.842 | 0.005804 |
| Fat:Carbohydrate | −6169.1 (1657.3) | −3.722 | 0.000385 |
| | | | |
| **Adjusted R-squared** | 0.7243 | | |
| **p-value** | < 2.2e−16 | | |

**Supplementary Table 4 – Statistical output for mixture models relating to plasma IgM**

| Model (Scheffé Polynomials) | Akaike Information Criterion | Degrees of freedom |
|---|---|---|
| 1 | 696.561 | 4 |
| 2 | 700.119 | 7 |
| 3 | 701.771 | 11 |
| 4 | 700.027 | 8 |
| Null | 693.555 | 2 |

**Supplementary Table 5 – 16S rRNA sequencing quality control**

| | Total reads | Reads post filtering | Reads post merging and chimera filtering | Reads retained |
|---|---|---|---|---|
| HP 1 | 135805 | 119366 | 97727 | 71.96% |
| HP 2 | 49009 | 43630 | 43301 | 88.35% |
| HP 3 | 120743 | 107426 | 91056 | 75.41% |
| HP 4 | 59370 | 51662 | 50927 | 85.78% |
| HP 5 | 71171 | 62355 | 61855 | 86.91% |
| HP 6 | 55324 | 49431 | 49288 | 89.09% |
| HP 7 | 24290 | 21876 | 21826 | 89.86% |
| HP 8 | 111569 | 96616 | 95494 | 85.59% |
| HC 1 | 89660 | 79158 | 78435 | 87.48% |
| HC 2 | 133887 | 119303 | 102883 | 76.84% |
| HC 3 | 52702 | 47334 | 46964 | 89.11% |
| HC 4 | 29954 | 26904 | 26437 | 88.26% |
| HC 5 | 121070 | 100002 | 94779 | 78.28% |
| HC 6 | 86078 | 71337 | 71144 | 82.65% |
| HC 7 | 69380 | 59366 | 59010 | 85.05% |
| HC 8 | 136477 | 117920 | 101334 | 74.25% |
| HF 1 | 44447 | 41330 | 40566 | 91.27% |
| HF 2 | 135418 | 126495 | 117867 | 87.04% |
| HF 3 | 13618 | 12835 | 12692 | 93.20% |
| HF 4 | 19284 | 18178 | 17971 | 93.19% |
| HF 5 | 23368 | 22069 | 21948 | 93.92% |
| HF 6 | 19495 | 18154 | 18134 | 93.02% |
| HF 7 | 120323 | 113333 | 103573 | 86.08% |
| HF 8 | 102020 | 95906 | 84963 | 83.28% |

**Supplementary Table 6 – Statistical output for the comparison of different dietary groups of different beta diversity distance metric, based on PERMANOVA test (permutation = 9999)**

| **PERMANOVA** | HP vs HC | HP vs HF | HC vs HF |
|---|---|---|---|
| Unweighted UniFrac | $R^2$= 0.18861<br>P= 0.005 | $R^2$= 0.34924<br>P= 2e-4 | $R^2$= 0.35927<br>P= 3e-4 |
| Weighted UniFrac | $R^2$= 0.45964<br>P= 2e-4 | $R^2$= 0.67843<br>P= 2e-4 | $R^2$= 0.81691<br>P= 2e-4 |
| Atchison's distance | $R^2$= 0.35152<br>P= 3e-04 | $R^2$= 0.53433<br>P= 1e-04 | $R^2$= 0.52121<br>P= 3e-04 |

**Supplementary Table 7 –Statistical output of ALDEx2 analysis comparing HP and HC at the genera level. CLR, centered log-ratio transformed; FDR, Benjamini–Hochberg corrected false discovery rate. Two-sided tests used.**

| Genus | Median CLR (HP) | Median CLR (HC) | Effect size | FDR (Welch's t test) | FDR (Wilcoxon test) |
|---|---|---|---|---|---|
| *Akkermansia* | 5.230 | -1.047 | -1.365 | 0.032 | 0.013 |
| *Lactococcus* | 7.187 | 3.426 | -1.481 | 0.024 | 0.009 |
| *Pseudomonas* | -4.515 | -6.937 | -0.439 | 0.404 | 0.427 |
| *Turicibacter* | -5.442 | -1.88 | 0.555 | 0.317 | 0.315 |
| *Barnesiella* | 7.097 | 7.503 | 0.257 | 0.628 | 0.773 |
| *Lactobacillus* | 9.757 | 7.297 | -0.683 | 0.185 | 0.278 |
| *Romboutsia* | -3.655 | 0.020 | 0.071 | 0.815 | 0.880 |
| *Allobaculum* | 7.151 | 8.922 | 1.063 | 0.061 | 0.050 |
| *Bifidobacterium* | 9.147 | 7.640 | -0.506 | 0.275 | 0.382 |
| *Olsenella* | 8.893 | 8.177 | -0.419 | 0.362 | 0.442 |
| *Clostridium_XIVa* | -2.954 | -5.438 | -0.439 | 0.416 | 0.437 |
| *Acetatifactor* | 1.263 | 2.133 | 0.582 | 0.165 | 0.194 |
| *Desulfovibrio* | 3.196 | 5.494 | 0.794 | 0.118 | 0.074 |
| *Parabacteroides* | -5.583 | -5.925 | -0.041 | 0.698 | 0.771 |
| *Coprobacter* | -0.637 | 2.605 | 0.732 | 0.145 | 0.175 |
| *Dorea* | 2.102 | 3.489 | 0.531 | 0.194 | 0.190 |
| *Sporobacter* | -6.180 | -4.408 | 0.346 | 0.440 | 0.541 |
| *Ruminococcus2* | -1.070 | -3.723 | -0.300 | 0.528 | 0.567 |
| *Clostridium_sensu_stricto* | 2.372 | -6.863 | -1.355 | 0.047 | 0.049 |
| *Enterorhabdus* | 1.809 | 2.222 | 0.128 | 0.660 | 0.833 |
| *Clostridium_IV* | -0.607 | -0.186 | 0.084 | 0.801 | 0.913 |
| *Mesorhizobium* | -0.669 | -0.841 | -0.057 | 0.828 | 0.905 |
| *Intestinimonas* | -4.610 | -5.968 | -0.253 | 0.609 | 0.679 |
| *Corynebacterium* | -6.419 | 0.715 | 0.818 | 0.166 | 0.228 |
| *Murimonas* | -2.197 | -6.755 | -0.743 | 0.235 | 0.214 |
| *Staphylococcus* | -3.963 | 2.500 | 0.642 | 0.211 | 0.168 |
| *Escherichia/Shigella* | -5.458 | -5.203 | 0.026 | 0.697 | 0.770 |
| *Ralstonia* | -4.315 | -3.492 | 0.156 | 0.635 | 0.743 |
| *Burkholderia* | -3.611 | -3.850 | 0.031 | 0.773 | 0.860 |
| *Enterococcus* | -6.467 | -2.824 | 0.633 | 0.276 | 0.286 |
| *Bacillus* | -6.061 | -2.534 | 0.627 | 0.265 | 0.297 |
| *Senegalimassilia* | -3.162 | -3.831 | -0.118 | 0.694 | 0.787 |

**Supplementary Table 8 –Statistical output of ALDEx2 analysis comparing HP and HF at the genera level. CLR, centered log-ratio transformed; FDR, Benjamini–Hochberg corrected false discovery rate. Two-sided tests used.**

| Genus | Median CLR (HP) | Median CLR (HF) | Effect size | FDR (Welch's t test) | FDR (Wilcoxon test) |
|---|---|---|---|---|---|
| *Akkermansia* | 5.201 | 8.460 | 1.823 | 0.002 | 0.003 |
| *Lactococcus* | 7.158 | 6.614 | -0.458 | 0.244 | 0.447 |
| *Pseudomonas* | -4.552 | 2.817 | 0.604 | 0.233 | 0.434 |
| *Turicibacter* | -5.315 | 9.107 | 4.267 | 0.000 | 0.001 |
| *Barnesiella* | 7.137 | 4.406 | -0.925 | 0.040 | 0.071 |
| *Lactobacillus* | 9.804 | 9.049 | -0.428 | 0.276 | 0.367 |
| *Romboutsia* | -3.374 | 7.604 | 1.943 | 0.006 | 0.001 |
| *Allobaculum* | 7.201 | -6.456 | -3.674 | 0.000 | 0.001 |
| *Bifidobacterium* | 9.221 | -5.819 | -4.804 | 0.000 | 0.001 |
| *Olsenella* | 8.948 | -6.443 | -4.254 | 0.000 | 0.001 |
| *Clostridium_XlVa* | -2.878 | 1.792 | 0.587 | 0.224 | 0.194 |
| *Acetatifactor* | 1.231 | 0.101 | -0.157 | 0.616 | 0.828 |
| *Desulfovibrio* | 3.181 | 3.842 | 0.119 | 0.672 | 0.671 |
| *Parabacteroides* | -5.452 | -3.252 | 0.407 | 0.370 | 0.395 |
| *Rhodococcus* | -6.354 | 0.442 | 0.699 | 0.213 | 0.272 |
| *Coprobacter* | -0.594 | 1.230 | 0.245 | 0.575 | 0.612 |
| *Dorea* | 2.128 | 1.273 | -0.194 | 0.752 | 0.603 |
| *Ruminococcus2* | -1.074 | -5.474 | -0.784 | 0.136 | 0.135 |
| *Clostridium_sensu_ stricto* | 2.420 | -6.603 | -1.184 | 0.041 | 0.048 |
| *Enterorhabdus* | 1.855 | 3.425 | 0.536 | 0.254 | 0.347 |
| *Clostridium_IV* | -0.654 | -2.718 | -0.290 | 0.552 | 0.515 |
| *Mesorhizobium* | -0.700 | 0.052 | 0.298 | 0.546 | 0.477 |
| *Intestinimonas* | -4.387 | -5.404 | -0.235 | 0.545 | 0.557 |
| *Murimonas* | -2.214 | -2.454 | 0.098 | 0.657 | 0.781 |
| *Staphylococcus* | -3.825 | -2.447 | 0.326 | 0.500 | 0.538 |
| *Escherichia/Shigell a* | -5.473 | -3.606 | 0.494 | 0.249 | 0.338 |
| *Ralstonia* | -4.262 | 0.189 | 0.518 | 0.314 | 0.250 |
| *Burkholderia* | -3.616 | -2.998 | 0.075 | 0.701 | 0.754 |
| *Enterococcus* | -6.275 | -2.906 | 0.500 | 0.329 | 0.310 |
| *Senegalimassilia* | -3.159 | -5.558 | -0.427 | 0.327 | 0.332 |

**Supplementary Table 9 –Statistical output of ALDEx2 analysis comparing HC and HF at the genera level. CLR, centered log-ratio transformed; FDR, Benjamini–Hochberg corrected false discovery rate. Two-sided tests used.**

| Genus | Median CLR (HC) | Median CLR (HF) | Effect size | FDR (Welch's t test) | FDR (Wilcoxon test) |
|---|---|---|---|---|---|
| *Akkermansia* | 5.201 | 8.460 | 1.823 | 0.002 | 0.003 |
| *Lactococcus* | 7.158 | 6.614 | -0.458 | 0.244 | 0.447 |
| *Pseudomonas* | -4.552 | 2.817 | 0.604 | 0.233 | 0.434 |
| *Turicibacter* | -5.315 | 9.107 | 4.267 | 0.000 | 0.001 |
| *Barnesiella* | 7.137 | 4.406 | -0.925 | 0.040 | 0.071 |
| *Lactobacillus* | 9.804 | 9.049 | -0.428 | 0.276 | 0.367 |
| *Romboutsia* | -3.374 | 7.604 | 1.943 | 0.006 | 0.001 |
| *Allobaculum* | 7.201 | -6.456 | -3.674 | 0.000 | 0.001 |
| *Bifidobacterium* | 9.221 | -5.819 | -4.804 | 0.000 | 0.001 |
| *Olsenella* | 8.948 | -6.443 | -4.254 | 0.000 | 0.001 |
| *Clostridium_XlVa* | -2.878 | 1.792 | 0.587 | 0.224 | 0.194 |
| *Acetatifactor* | 1.231 | 0.101 | -0.157 | 0.616 | 0.828 |
| *Desulfovibrio* | 3.181 | 3.842 | 0.119 | 0.672 | 0.671 |
| *Parabacteroides* | -5.452 | -3.252 | 0.407 | 0.370 | 0.395 |
| *Rhodococcus* | -6.354 | 0.442 | 0.699 | 0.213 | 0.272 |
| *Coprobacter* | -0.594 | 1.230 | 0.245 | 0.575 | 0.612 |
| *Dorea* | 2.128 | 1.273 | -0.194 | 0.752 | 0.603 |
| *Ruminococcus2* | -1.074 | -5.474 | -0.784 | 0.136 | 0.135 |
| *Clostridium_sensu_ stricto* | 2.420 | -6.603 | -1.184 | 0.041 | 0.048 |
| *Enterorhabdus* | 1.855 | 3.425 | 0.536 | 0.254 | 0.347 |
| *Clostridium_IV* | -0.654 | -2.718 | -0.290 | 0.552 | 0.515 |
| *Mesorhizobium* | -0.700 | 0.052 | 0.298 | 0.546 | 0.477 |
| *Intestinimonas* | -4.387 | -5.404 | -0.235 | 0.545 | 0.557 |
| *Murimonas* | -2.214 | -2.454 | 0.098 | 0.657 | 0.781 |
| *Staphylococcus* | -3.825 | -2.447 | 0.326 | 0.500 | 0.538 |
| *Escherichia/Shigell a* | -5.473 | -3.606 | 0.494 | 0.249 | 0.338 |
| *Ralstonia* | -4.262 | 0.189 | 0.518 | 0.314 | 0.250 |
| *Burkholderia* | -3.616 | -2.998 | 0.075 | 0.701 | 0.754 |
| *Enterococcus* | -6.275 | -2.906 | 0.500 | 0.329 | 0.310 |
| *Senegalimassilia* | -3.159 | -5.558 | -0.427 | 0.327 | 0.332 |

**Supplementary Table 10 – Concentration of small intestine luminal metabolites detected by nuclear magnetic resonance (NMR). Concentrations are expressed as mM per mg of SI content (SD).**

| | HP | HC | HF |
|---|---|---|---|
| **2-Aminobutyrate** | 1.076 (2.789) | 0.753 (0.893) | 0.624 (1.205) |
| **Acetate** | 0.792 (0.199) | 0.633 (0.127) | 0.330 (0.159) |
| **Alanine** | 3.981 (1.553) | 2.561 (1.291) | 0.000 (1.365) |
| **Choline** | 1.208 (0.357) | 1.434 (0.272) | 1.068 (0.148) |
| **Fumarate** | 0.094 (0.009) | 0.066 (0.038) | 0.000 (0.009) |
| **Glutamate** | 5.568 (1.792) | 3.293 (0.462) | 2.850 (1.271) |
| **Glycine** | 1.335 (0.309) | 1.054 (0.553) | 1.252 (0.361) |
| **Homoserine** | 0.000 (1.212) | 2.054 (0.877) | 1.703 (0.466) |
| **Isoleucine** | 3.090 (0.571) | 2.070 (0.734) | 1.408 (0.503) |
| **Lactose** | 2.468 (0.628) | 1.450 (0.295) | 0.946 (0.152) |
| **Leucine** | 9.563 (1.047) | 5.229 (1.082) | 4.309 (0.857) |
| **Lysine** | 3.674 (1.803) | 3.284 (0.877) | 2.852 (0.463) |
| **Malonate** | 0.366 (0.366) | 0.310 (0.310) | 0.291 (0.291) |
| **Methionine** | 2.791 (0.425) | 1.393 (0.711) | 1.219 (0.468) |
| **Pyruvate** | 0.133 (0.242) | 0.264 (0.129) | 0.125 (0.119) |
| **Serine** | 1.945 (0.942) | 1.227 (0.832) | 0.000 (0.431) |
| **Succinate** | 0.039 (0.005) | 0.017 (0.011) | 0.013 (0.008) |
| **Valine** | 5.303 (1.046) | 3.182 (0.688) | 2.429 (0.800) |

**Supplementary Table 11 – Concentration of cecal content metabolites detected by nuclear magnetic resonance (NMR). Concentrations are expressed as mM per mg of cecal content (SD).**

| | HP | HC | HF |
|---|---|---|---|
| **Acetate** | 23.21 (4.367) | 35.50 (15.36) | 14.87 (2.479) |
| **Alanine** | 0.4942 (0.6841) | 0.5355 (0.6802) | 0.2778 (0.2390) |
| **Butyrate** | 3.558 (0.8711) | 8.759 (3.395) | 2.027 (1.026) |
| **Glutamate** | 4.083 (1.991) | 2.808 (2.193) | 1.026 (1.109) |
| **Isoleucine** | 0.6867 (0.3561) | 0.1078 (0.2042) | 0 (0) |
| **Lactate** | 1.457 (0.8441) | 1.119 (1.143) | 1.909 (1.849) |
| **Leucine** | 1.597 (0.9185) | 0.5091 (0.4334) | 0.1735 (0.2790) |
| **Lysine** | 0.8053 (0.9037) | 0.6552 (0.6331) | 0 (0) |
| **Malonate** | 0.3504 (0.1826) | 0.3163 (0.1561) | 0.1881 (0.1885) |
| **Methionine** | 0.2958 (0.2621) | 0.1782 (0.1760) | 0.03506 (0.07632) |
| **Propionate** | 5.771 (1.606) | 2.777 (0.8856) | 3.297 (0.6736) |
| **Pyruvate** | 1.445 (0.6071) | 0.6418 (0.2915) | 0.4338 (0.1698) |
| **Succinate** | 0.7279 (0.2722) | 0.2428 (0.08354) | 0.1241 (0.07370) |
| **Valine** | 0.8662 (0.4700) | 0.4675 (0.3449) | 0.03995 (0.1130) |

**Supplementary Table 12 – Antibodies used for flow cytometry**

| Target | Conjugate | Clone | Dilution | Manufacturer |
|---|---|---|---|---|
| CD45 | BV785 | 30-F11 | 1:400 | BioLegend |
| CD95 | APC | SA367H8 | 1:200 | BioLegend |
| GL-7 | FITC | GL7 | 1:200 | BioLegend |
| B220 | VioGreen | REA755 | 1:50 | Miltenyi Biotec |
| B220 | APC | RA3-6B2 | 1:200 | BioLegend |
| IgA | PE | 11-44-2 | 1:200 | eBioscience |
| CD4 | PerCP | RM4-5 | 1:200 | BioLegend |

**Supplementary Table 13 – Primers used in this study and their sequence**

Pre-designed and validated primers used in this study. All primers were purchased from Sigma-Aldrich (KiCqStart SYBR® Green primers).

| Gene target | Specie | Forward (5'-3') | Reverse (5'-3') |
|---|---|---|---|
| *Pigr* | Mouse | AAGAACTCCAGAGATTTGGG | GTGGTAGTCACGATTTCATC |
| *Ccl28* | Mouse | GAGGTGTCTCATCATGTTTC | ATACGTTTTCTCTGCCATTC |
| *Tnfsf13* | Mouse | TCTATAGTCAGGTCCTGTTTC | GGCATACTTCTGATACATCG |
| *Baff* | Mouse | ATCTACAGCCAGGTTCTATAC | AGCTGAATCTCATCTCCTTC |
| *Tgfb* | Mouse | GGATACCAACTATTGCTTCAG | TGTCCAGGCTCCAAATATAG |
| *Tslp* | Mouse | CCTGAAACTGAGAGAAATGAC | ACACCCTTAGTATTCTGTCC |
| *Il10* | Mouse | AAGGGTTACTTGGGTTGCCA | AAATCGATGACAGCGCCTCAG |
| *Il4* | Mouse | CTGGATTCATCGATAAGCTG | TTTGCATGATGCTCTTTAGG |
| *Muc2* | Mouse | ATTCTGAAGCCTGGGGAGAT | GAAGTCGGGACAGGTGATGT |
| *Tjp1* | Mouse | GTCTGCCATTACACGGTCCT | GGCTTAAGTCCAGGGGAGTC |
| *Ocln* | Mouse | CGGTACAGCAGCAATGGTAA | CTCCCCACCTGTCGTGTAGT |
| *Tlr4* | Mouse | GATCAGAAACTCAGCAAAGTC | TGTTTCAATTTCACACCTGG |
| *Rpl13a* | Mouse | ATCCCTCCACCCTATGACAA | GCCCCAGGTAAGCAAACTT |
| *GAPDH* | Human | GAAGGTGAAGGTCGGAGTCA | CAGAGTTAAAAGCAGCCCTGG |
| *CCL28* | Human | GAGGTGTCTCATCATGTTTC | ATACGTTTTCTCTGCCATTC |
| *APRIL* | Human | CAGGTGTCTTCCATTTACAC | TGGAGAGAGGTTAAGTTTCG |
| *PIGR* | Human | GACCGAGTTTCAATCAGAAG | TTGTCATTGGCTCCAAATTC |

**Supplementary Figure 1**

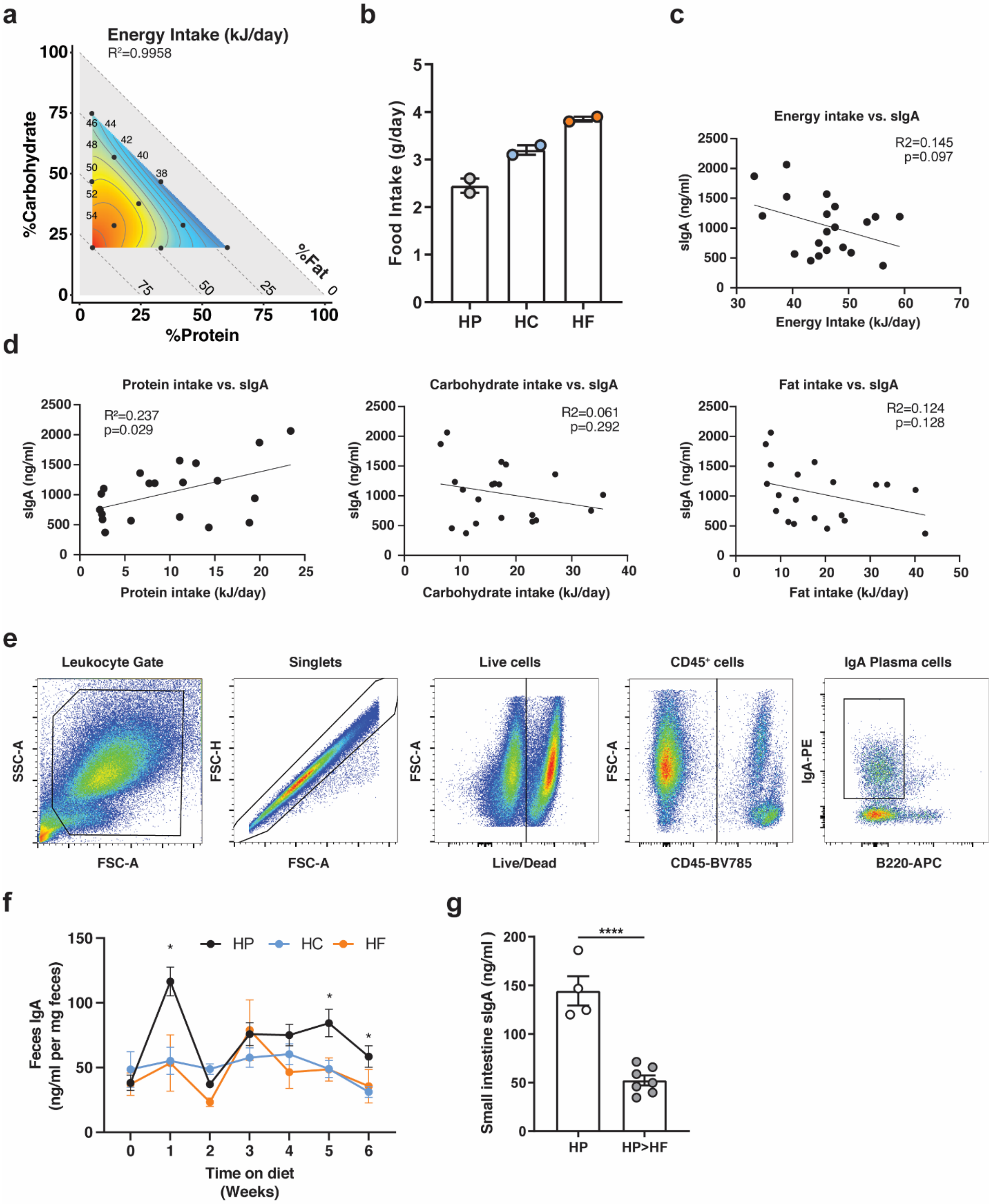

Supplementary Figure 1: Mice were fed on one of 10 diets encompassing a macronutrient range of 5-60% protein, 20-75% carbohydrate, and 20-75% fat for 6 weeks. (a) Contribution of macronutrient composition to average total daily energy intake (kJ/day) (averaged cage data) was modelled by Scheffé mixture model and represented on a right-angled mixture triangle. 3 component mixture model represented on a right-angled mixture triangle comprising of carbohydrate (y-axis), protein (x-axis) and fat (hypotenuse) with average total daily energy intake (kJ/day) as the response variable. Red represents higher average total daily energy intake while blue represents lower average total daily energy intake (kJ/day) in the nutrient mixture space. Each dot represents one of the 10 diets used for modelling response surface. (b) Average food intake (each point represents averaged cage data of n=4 mice per cage) of mice fed on a high-protein (HP), high-carbohydrate (HC) or high-fat (HF) diet. Linear regression of (c) total energy intake vs. sIgA levels as well as (d) protein, carbohydrate and fat eaten vs. sIgA levels were performed by simple linear regression analysis. (e) Gating strategy for the identification of $B220^{-}IgA^{+}$ IgA plasma cells by flow cytometry. (f) Mice were fed on a HP, HC or HF diet for 6 weeks and feces were collected weekly for determination of feces IgA by ELISA (n=6 mice per group for Week 0-2 and n=10 mice per group for Week 3-6). (g) Mice were fed on a HP diet for 6 weeks or fed on a HP diet for 6 weeks then switched to a HF diet for an additional 6 weeks and sIgA determined by ELISA (n=4 mice per group for HP and n=7 mice per group for HP>HF; p = <0.0001 by two-tailed unpaired t-test). Data are represented as mean ± SEM. Results represent n=3 independent (a-b), n=2 (c-e) and n=1 experiments (f-g).

**Supplementary Figure 2**

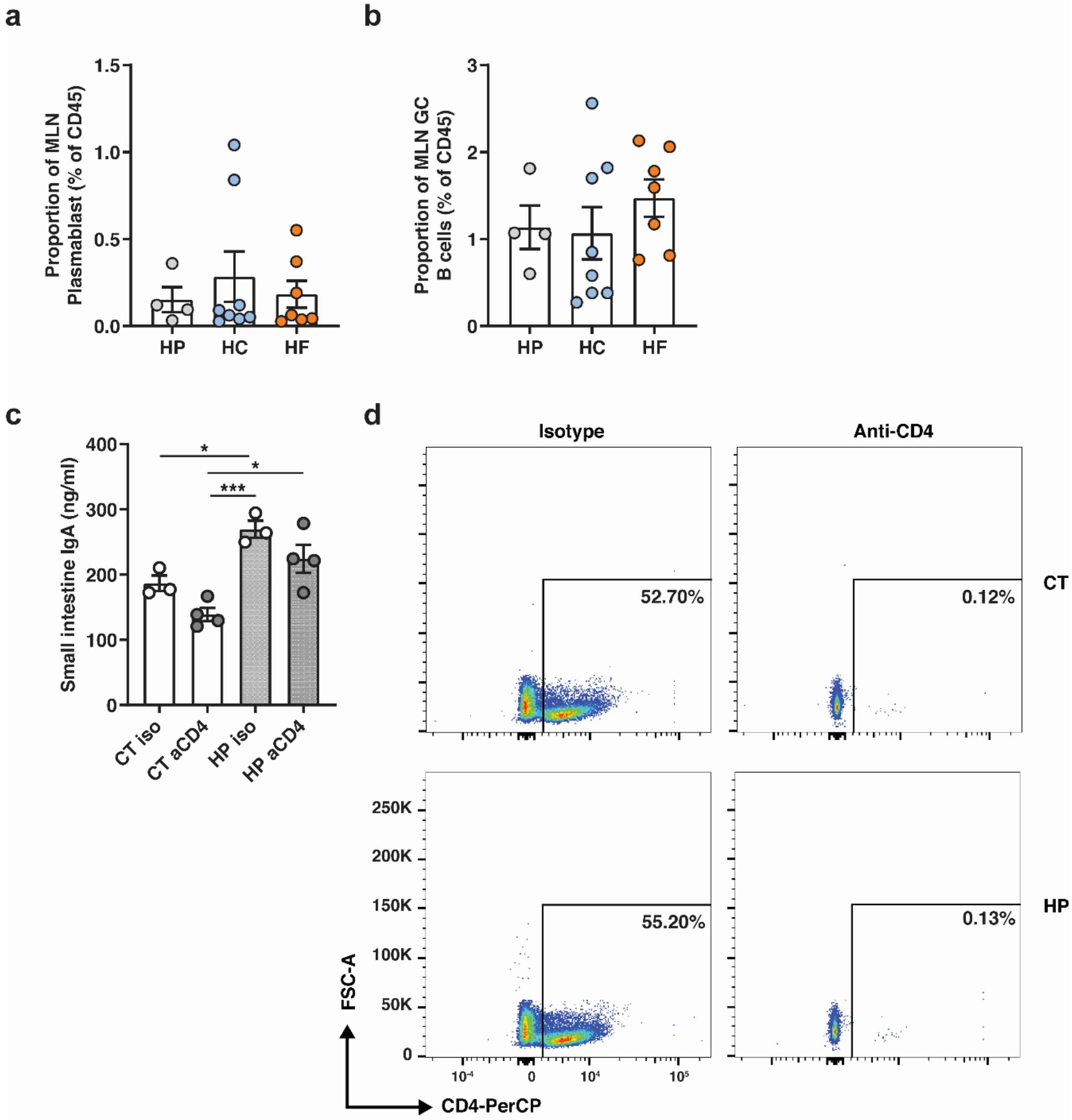


Supplementary Figure 2: (a) Proportion of B220⁺IgA⁺ plasmablast and (b) CD95⁺GL7⁺ germinal centre B cells in the mesenteric lymph nodes (n=4, n=7 and n=8 mice per diet for HP, HC and HF diet respectively) were determined by flow cytometry from mice fed on either a high-protein (HP), high-carbohydrate (HC) or high-fat (HF) diet for 6 weeks. (c) Mice were administered 200µg of anti-CD4 (GK1.5) or isotype control (LTF-2) antibody one day prior to either HP or control (AIN93G) diet feeding. Antibody treatment was administered twice weekly and small intestine sIgA determined 6 weeks later (n=3 mice per group for isotype control and n=4 mice per group for anti-CD4 treated groups; CT iso vs. HP iso p=0.0276, CT aCD4 vs HP iso p=0.0008, CT aCD4 vs. HP aCD4 p=0.01 by ordinary one-way ANOVA followed by Tukey's multiple comparisons test). Data are represented as mean ± SEM. (d) Representative flow cytometry plot demonstrating the successful systemic depletion of CD4⁺ lymphocytes by flow cytometry. Results represent n=2 independent (a-b) and n=1 independent experiment (c-d).

**Supplementary Figure 3**

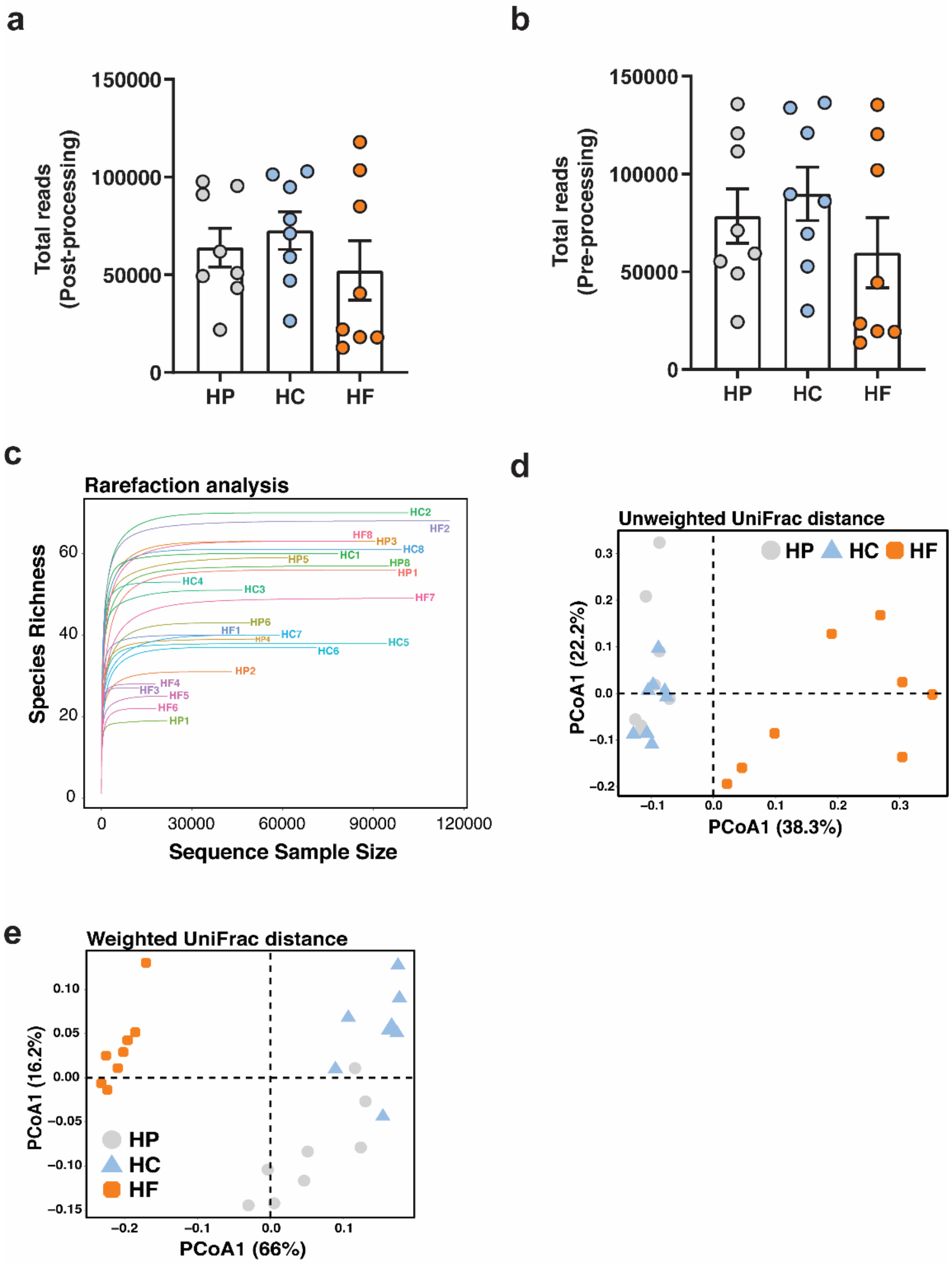


Supplementary Figure 3: DNA from luminal content was extracted for 16S rRNA DNA sequencing from mice fed on a high-protein (HP), high-carbohydrate (HC) or high-fat (HF) diet for 6 weeks (n=8 per group). The total number of reads per samples (a) post and (b) pre-processing were quantified and no statistical significance between groups were found (Kruskal-Wallis test). Data are represented as mean ± SEM. (c) Rarefaction analysis was performed for each sample demonstrating that sequencing depth was sufficient to uncover all unique taxa (ASV) present in each sample. (d-e) Differences in the structure of the small intestinal microbiota communities were determined by principal coordinate analysis (PCoA) of both (d) Unweighted and (e) Weighted UniFrac distances. Results represent n=1 independent experiment.

**Supplementary Figure 4**

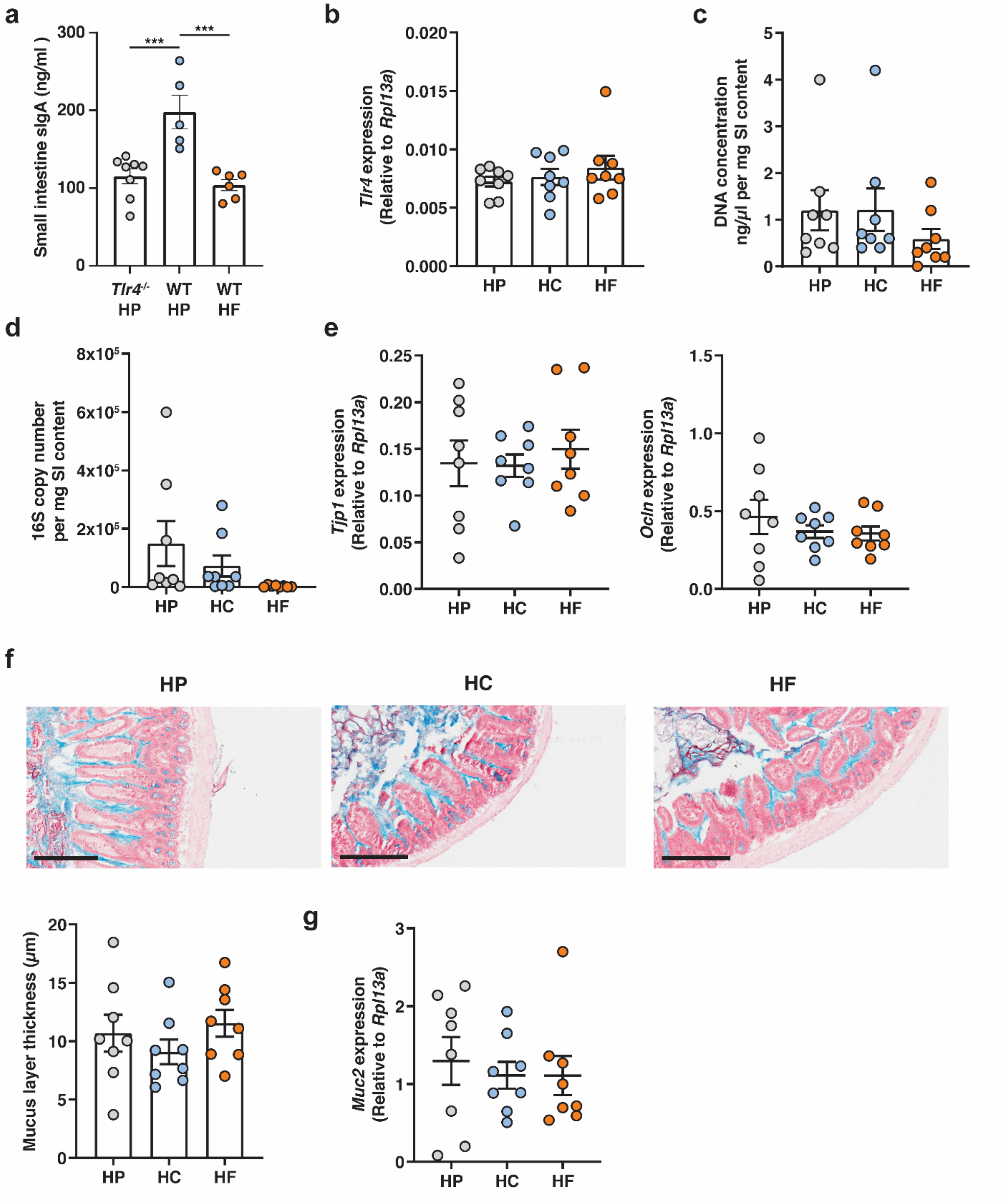

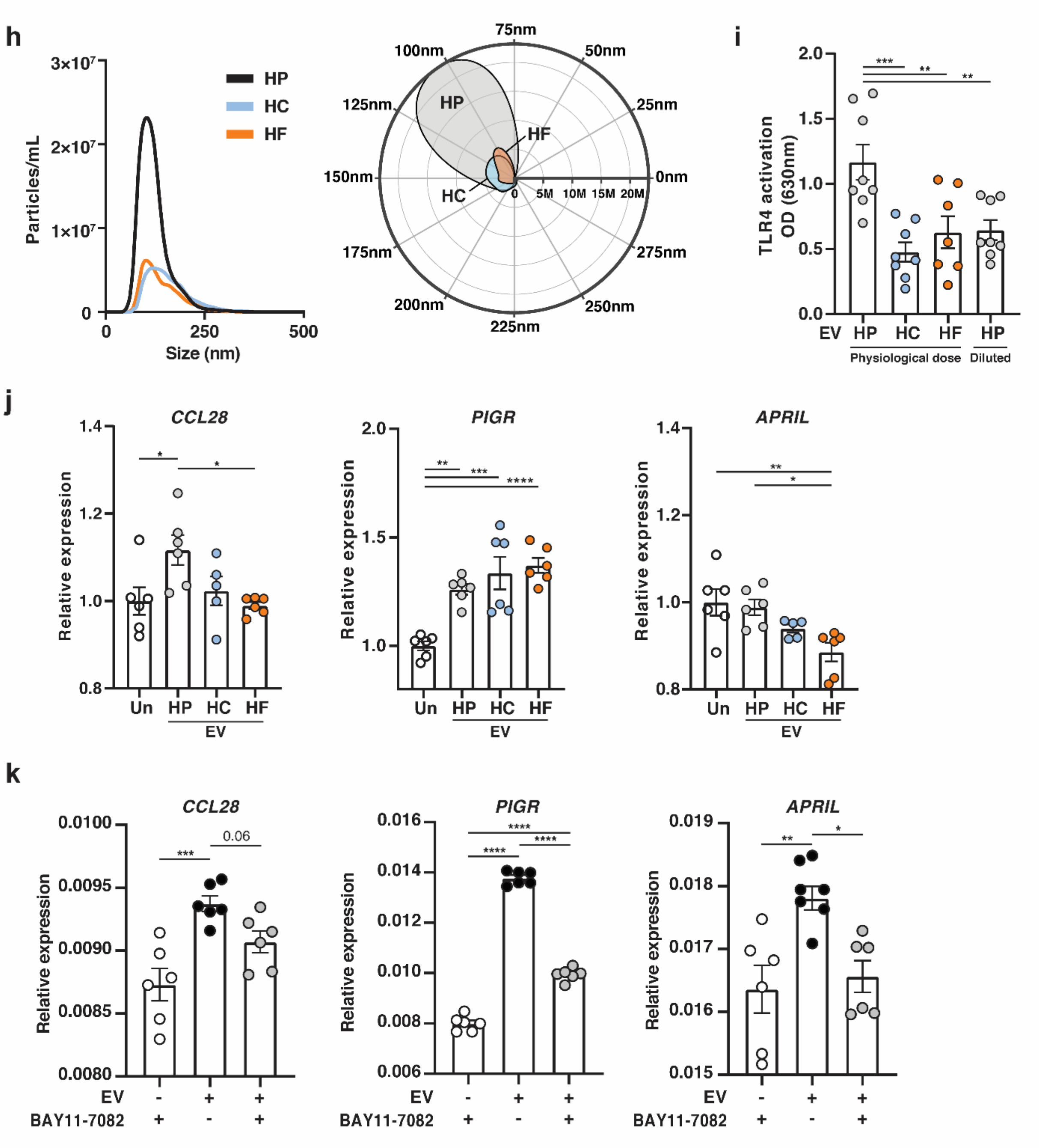


Supplementary Figure 4: (a) *Tlr4*$^{-/-}$ or wild type (WT) mice were fed on a high-protein (HP) diet, or WT mice fed on a high-fat (HF) diet for 6 weeks and sIgA levels were determined by ELISA (n=8, n=5 and n=6 mice per group for *Tlr4*$^{-/-}$ HP, WT HP and WT HF respectively; *Tlr4*$^{-/-}$ HP vs. WT HP p=0.008, WT HP vs. WT HF p=0.0004). Wild type mice were fed on either a high-protein (HP), high-carbohydrate (HC) or high-fat (HF) diet for 6 weeks and small intestine gene expression of (b) *Tlr4*, (e) *Tjp1* and *Ocln* and (g) *Muc2* was determined by qPCR (n=8 mice per group). Their small intestine contents were collected for DNA extraction and (c) DNA concentration was quantified by the Qubit™ dsDNA HS Assay Kit and (d) total 16S copy number by qPCR (n=8 mice per group). Wild type mice were fed on either a high-protein (HP), high-carbohydrate (HC) or high-fat (HF) diet for 6 weeks and (f) average mucus layer thickness was determined by histological analysis of Alcian blue-stained small intestine sections with the scale bar representing

100µm. (n=8 mice per group) (h) Cecum microbiota-derived extracellular vesicles (EV) were characterised by Nanoparticle Tracking Analysis (n=2 per diet pooled from n=4 mice each) and represented as an XY plot (left): 0-500nm vs. particle number/mL, or polar plot (right): angular axis represents particle size between 0-300nm and radial axis represents particle concentration in number/mL). (i) Cecum microbiota-derived EV were incubated at physiological ratio (approximately 3:1:1 of HP:HC:HF; n=8,n=8 and n=7 mice per group respectively), or at 1:1:1 ratio (HP diluted, n=8 mice per group) overnight with HEK-Blue TLR4 cell line and TLR activation measured at 630nm (HP vs. HC p=0.0004, HP vs. HF p=0.0072, HP vs. HP diluted p=0.007). (j) HT-29 were stimulated with cecum microbiota-derived EV isolated from HP ($3x10^9$ EV per well) or HC/HF ($1x10^9$ EV per well) fed animals for 16 hours and expression of CCL28 (Un vs. HP p=0.0372, HP vs. HF p=0.0213), PIGR (Un vs. HP p=0.0027, Un vs. HC p=0.0002, Un vs. HF p=<0.0001) and APRIL (Un vs. HF p=0.0067, HP vs. HF p=0.0156) were quantified by qPCR (n=6 wells per condition) and as well as (k) in the presence or absence of 10µM of NF-κB inhibitor BAY 11-7082, or with BAY 11-7082 alone and expression of CCL28 (-EV+Bay11-7082 vs. +EV-BAY11-7082 p=0.008), PIGR (-EV+Bay11-7082 vs. +EV-BAY11-7082 p=<0.0001, -EV+Bay11-7082 vs. +EV+Bay11-7082 p=<0.0001, +EV-BAY11-7082 vs. +EV+Bay11-7082 p=<0.0001) and APRIL (-EV+Bay11-7082 vs. +EV-BAY11-7082 p=0.0069, +EV-BAY11-7082 vs. +EV+BAY11-7082 p=0.0188) were quantified by qPCR (n=6 wells per condition). Data are represented as mean ± SEM. Results represent n=2 (b-e, g-k) and n=1 independent experiment(s) (a, f). *p<0.05, **<0.01, ***<0.001, ****<0.0001 by ordinary one-way ANOVA followed by Tukey's multiple comparisons test.

**Supplementary Figure 5**

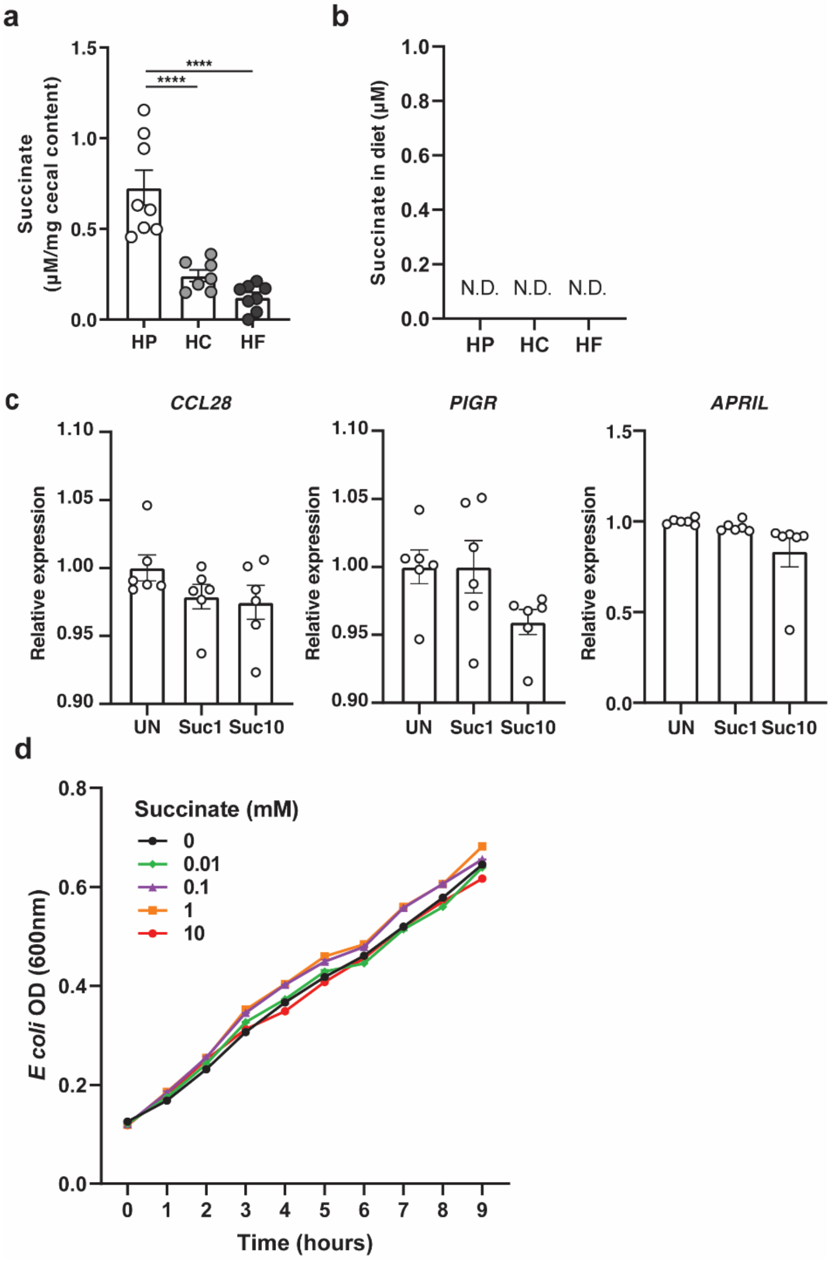


Supplementary Figure 5: (a) Levels of succinate in the cecum were quantified by NMR spectroscopy from mice fed on a high-protein (HP), high-carbohydrate (HC) or high-fat (HF) diet for 6 weeks (n=7 and n=8 mice per diet for HC and HP/HF diet respectively; HP vs. HC p=<0.0001, HP vs. HF p= p=<0.0001). (b) Levels of succinate in food pellet of the HP, HC or HF diet was quantified by NMR spectroscopy (n=2 pellet from each diet were analyzed to confirm undetectable levels of succinate). (c) HT29 were stimulated overnight with vehicle control (PBS), or 1mM or 10mM of succinate and gene expression levels of CCL28, PIGR and APRIL quantified by qPCR (n=6 wells per condition). (d) Growth of *E coli,* cultured with the indicated concentrations of succinate, was quantified by spectrophotometry (OD 600nm). Data are represented as mean ± SEM. Results represent n=2 (d) and n=1 independent experiments (a-c). ****p<0.0001 by ordinary one-way ANOVA followed by Tukey's multiple comparisons test.
