# Supplementary material for: Dietary protein increases T-cell-independent sIgA production through changes in gut microbiota-derived extracellular vesicles
Source: Nat Commun. 2022 Jul 27;13:4336. doi: 10.1038/s41467-022-31761-y (PMC9329401; doi:10.1038/s41467-022-31761-y)
Supplement: Supplementary file 3 — Reporting Summary [file 41467_2022_31761_MOESM3_ESM.pdf]

nature portfolio

Corresponding author(s): Laurence Macia

Last updated by author(s): Jun 2, 2022

Reporting Summary

Nature Portfolio wishes to improve the reproducibility of the work that we publish. This form provides structure for consistency and transparency in reporting. For further information on Nature Portfolio policies, see our Editorial Policies and the Editorial Policy Checklist.

Statistics

For all statistical analyses, confirm that the following items are present in the figure legend, table legend, main text, or Methods section.

n/a	Confirmed	
☐	☒	The exact sample size (*n*) for each experimental group/condition, given as a discrete number and unit of measurement
☐	☒	A statement on whether measurements were taken from distinct samples or whether the same sample was measured repeatedly
☐	☒	The statistical test(s) used AND whether they are one- or two-sided<br>*Only common tests should be described solely by name; describe more complex techniques in the Methods section.*
☒	☐	A description of all covariates tested
☐	☒	A description of any assumptions or corrections, such as tests of normality and adjustment for multiple comparisons
☐	☒	A full description of the statistical parameters including central tendency (e.g. means) or other basic estimates (e.g. regression coefficient) AND variation (e.g. standard deviation) or associated estimates of uncertainty (e.g. confidence intervals)
☐	☒	For null hypothesis testing, the test statistic (e.g. *F*, *t*, *r*) with confidence intervals, effect sizes, degrees of freedom and *P* value noted<br>*Give P values as exact values whenever suitable.*
☒	☐	For Bayesian analysis, information on the choice of priors and Markov chain Monte Carlo settings
☒	☐	For hierarchical and complex designs, identification of the appropriate level for tests and full reporting of outcomes
☒	☐	Estimates of effect sizes (e.g. Cohen's *d*, Pearson's *r*), indicating how they were calculated

Our web collection on statistics for biologists contains articles on many of the points above.

Software and code

Policy information about availability of computer code

Data collection	Flow cytometry data was acquired on an LSRII (BD) using BD FACS DIVA software v8.0.2. Particle data was acquired with the Nanosight300 with the NTA software v3.2. qPCR data was acquired using the LightCycler 480 Instrument and the LightCycler software v1.5.0. Immunohistochemistry data was captured with an Olympus BX51 Microscope using the cellSens Standard v1.12 software. 16S rRNA gene sequencing data was acquired on a Illumina MiSeq. Optical density and fluoresence measurements were acquired using the TECAN Infinite M1000 PRO using the Tecan i-control v1.10.4.0 software.
Data analysis	Microsoft Excel (Office 365 build 2203)<br>Flowjo v10.4.2<br>Graphpad prism 9<br>Living Image 4.5<br>Aperio ImageScope v12.3.3<br>FIJI v1.52p<br>R Software 3.6.1 with RStudio 1.4.1717<br>The following R packages were used: mixexp (1.2.5), plyr (1.8.6), scales (1.1.1), directlabels (2021.1.13), sp (1.4-5), readr (1.4.0), vegan (2.5-7), zCompositions (1.3.4), phyloseq (1.32.0), microbiome (1.10.0), dada2 (1.16.0), ALDEx2 (1.20.0), metagMisc (0.0.4), ranacapa (0.1.0), phangorn (2.5.5), dada2 (1.12.1)

For manuscripts utilizing custom algorithms or software that are central to the research but not yet described in published literature, software must be made available to editors and reviewers. We strongly encourage code deposition in a community repository (e.g. GitHub). See the Nature Portfolio guidelines for submitting code & software for further information.

Data

Policy information about availability of data

All manuscripts must include a data availability statement. This statement should provide the following information, where applicable:

- Accession codes, unique identifiers, or web links for publicly available datasets
- A description of any restrictions on data availability
- For clinical datasets or third party data, please ensure that the statement adheres to our policy

DNA sequencing data were deposited in the European Nucleotide Archive under accession number PRJEB39583 [https://www.ebi.ac.uk/ena/browser/view/PRJEB39583]. Source data are provided with this paper. Other data supporting the findings of this study are available from the corresponding author upon request.

Field-specific reporting

Please select the one below that is the best fit for your research. If you are not sure, read the appropriate sections before making your selection.

☒ Life sciences ☐ Behavioural & social sciences ☐ Ecological, evolutionary & environmental sciences

For a reference copy of the document with all sections, see nature.com/documents/nr-reporting-summary-flat.pdf

Life sciences study design

All studies must disclose on these points even when the disclosure is negative.

Sample size	No sample size calulation was performed, sample size were determined based on prior similar experiments (Tan et al, 2017, Cell Reports) and according to the principles of the 3Rs (Replacement, Reduction and Refinement).
Data exclusions	No successfully acquired data was excluded.
Replication	The number of independent replication of experiments is indicated in the corresponding figure legend for each figure.
Randomization	Mice were age and sex-matched for each experiment, and beddings were mixed between cages to normalise microbiome composition before beginning experiments. Each cage of mice were randomized to different experimental groups, but individual mice were not randomized to experimental group as male mice from different cages cannot be co-housed due to fighting post-weaning.
Blinding	For histological analysis of mucus thickness layer, images were scored/analyzed blinded to the study design. Histological analysis of colon sections from DDS-induced colitis mice were scored by at least 2 independent researcher who were not blinded to the study design but were blinded to the samples (images were captured and filenames deidentified for scoring). No blinding was performed for analysis of in vitro experiments as well as flow cytometry experiments as the data analysis was performed using standard operating procedure such as pre-defined gating strategies and the data as well as its analysis are quantitative in nature and not subjective.

Reporting for specific materials, systems and methods

We require information from authors about some types of materials, experimental systems and methods used in many studies. Here, indicate whether each material, system or method listed is relevant to your study. If you are not sure if a list item applies to your research, read the appropriate section before selecting a response.

Materials & experimental systems

n/a	Involved in the study	
☐	☒	Antibodies
☐	☒	Eukaryotic cell lines
☒	☐	Palaeontology and archaeology
☐	☒	Animals and other organisms
☒	☐	Human research participants
☒	☐	Clinical data
☒	☐	Dual use research of concern

Methods

n/a	Involved in the study	
☒	☐	ChIP-seq
☐	☒	Flow cytometry
☒	☐	MRI-based neuroimaging

Antibodies

Antibodies used	The following anti-mouse antibodies were used in this study: CD45-BV785 (30-F11) Cat#103149, CD95-APC (SA367H8) Cat#152604, GL-7-FITC (GL7) Cat#144603, B220-APC (RA3-6B2) Cat#103212, CD4-PerCP (RM4-5) Cat#100538 from BioLegend, IgA-PE (11-44-2) Cat#12-5994-81 from eBioscience, B220-VioGreen (REA755) Cat#130-110-852 from Miltenyi-Biotec
Validation	All antibodies are from commercial sources and used according to the manufacturer's instruction. Validation data are available from

the manufacturer's website (BioLegend, eBioscience and Miltenyi-Biotec):

CD45-BV785 (30-F11) Cat#103149
https://www.biolegend.com/en-us/products/brilliant-violet-785-anti-mouse-cd45-antibody-10636

CD95-APC (SA367H8) Cat#152604
https://www.biolegend.com/en-us/products/apc-anti-mouse-cd95-fas-antibody-13906

GL-7-FITC (GL7) Cat#144603
https://www.biolegend.com/en-us/products/fitc-anti-mouse-human-gl7-antigen-t-and-b-cell-activation-marker-antibody-8284

B220-APC (RA3-6B2) Cat#103212
https://www.biolegend.com/en-us/products/apc-anti-mouse-human-cd45r-b220-antibody-442

CD4-PerCP (RM4-5) Cat#100538
https://www.biolegend.com/en-us/products/percp-anti-mouse-cd4-antibody-4229

IgA-PE (11-44-2) Cat#12-5994-81
https://www.thermofisher.com/antibody/product/IgA-Antibody-clone-11-44-2-Monoclonal/12-5994-81

B220-VioGreen (REA755) Cat#130-110-852
https://www.miltenyibiotec.com/AU-en/products/cd45r-b220-antibody-anti-mouse-reafinity-rea755.html#percp-vio-700:30-ug-in-200-ul

Eukaryotic cell lines

Policy information about cell lines

Cell line source(s)	HT29 was obtained from ATCC. HEK-Blue mTLR4 was obtained from InvivoGen
Authentication	Authentication of cell line was not performed for HT29 cellline. HEK-Blue mTLR4 cell line was validated functionally, based on its reporter activity against TLR4 ligand using LPS as a positive control.
Mycoplasma contamination	Cell lines were routinely tested negative for mycoplasma contamination
Commonly misidentified lines (See ICLAC register)	No commonly misidentified lines were used in this study

Animals and other organisms

Policy information about studies involving animals; ARRIVE guidelines recommended for reporting animal research

Laboratory animals	Male C57BL/6 mice (6 weeks of age) were purchased from Animal Bioresource (NSW, Australia) and maintained under specific pathogen-free conditions, with a 12h light/dark cycle maintained at 22 degrees celcius, 50% humidity. Animals were housed in cages with non-edible compressed cotton fibre (iso-PADS) bedding.
Wild animals	This study did not involve the use of wild animals
Field-collected samples	This study did not collect samples in the field
Ethics oversight	All animals were housed in the animal facility of the Charles Perkins Centre. All experiments were performed in accordance with protocols approved by the University of Sydney Animal Ethics Committee.

Note that full information on the approval of the study protocol must also be provided in the manuscript.

Flow Cytometry

Plots

Confirm that:

- [x] The axis labels state the marker and fluorochrome used (e.g. CD4-FITC).
- [x] The axis scales are clearly visible. Include numbers along axes only for bottom left plot of group (a 'group' is an analysis of identical markers).
- [x] All plots are contour plots with outliers or pseudocolor plots.
- [x] A numerical value for number of cells or percentage (with statistics) is provided.

Methodology

Sample preparation	Single-cell suspensions were prepared by mechanical disruption of tissues through a 100µm filter. For isolation of intestinal

lamina propria cells, mesenteric fat and Peyer's patches were first resected from small intestine tissues, then tissues washed with PBS and cut into 1cm pieces and incubated in pre-digestion buffer (HBSS containing 10% FBS, 10mM HEPES, 5mM EDTA) for 40min at 37oC at 200rpm in an orbital incubator. Tissues were washed twice with PBS then cut into <1mm pieces and incubated in digestion buffer (RPMI containing 10% FBS, 10mM HEPES, 2.5mg/ml collagenase type IV (Gibco)) for 60min at 37oC at 200rpm in an orbital incubator and lymphocytes were enriched via a 40/80% Percoll gradient (GE Healthcare).

Instrument	BD LSR II flow cytometer
Software	BD FACSDiva software v8.0.2 was used for sample acquisition. FlowJo v10 was used for data analysis.
Cell population abundance	For analysis of B cells from the Peyer's patches and mesenteric lymph nodes, at least 30,000 B220+ cells was acquired for analysis. For analysis of small intestinal lamina propria lymphocytes all events were acquired for analysis.
Gating strategy	For analysis, a FSC/SSC gate was first employed to gate on cells that represents lymphocytes. A singlet gate was then applied (FSC-A vs. FSC-H), followed by exclusion of dead cells (FSC-A vs. negative Live/Dead UV staining). Following this, total lymphocyte was gated (FSC-A vs. CD45 positive staining). This population was then used to identify various cell type as described in the manuscript.

☒ Tick this box to confirm that a figure exemplifying the gating strategy is provided in the Supplementary Information.
